# microRNAs in mycobacterial disease: friend or foe?

**DOI:** 10.3389/fgene.2014.00231

**Published:** 2014-07-15

**Authors:** Manali D. Mehta, Philip T. Liu

**Affiliations:** ^1^Department of Microbiology, Immunology and Molecular Genetics, University of California at Los AngelesLos Angeles, CA, USA; ^2^Orthopaedic Hospital Research Center, University of California at Los AngelesLos Angeles, CA, USA; ^3^Division of Dermatology, Department of Medicine, David Geffen School of Medicine, University of California at Los AngelesLos Angeles, CA, USA

**Keywords:** tuberculosis, mycobacteria, leprosy, microRNA, vitamin D

## Abstract

As the role of microRNA in all aspects of biology continues to be unraveled, the interplay between microRNAs and human disease is becoming clearer. It should come of no surprise that microRNAs play a major part in the outcome of infectious diseases, since early work has implicated microRNAs as regulators of the immune response. Here, we provide a review on how microRNAs influence the course of mycobacterial infections, which cause two of humanity’s most ancient infectious diseases: tuberculosis and leprosy. Evidence derived from profiling and functional experiments suggests that regulation of specific microRNAs during infection can either enhance the immune response or facilitate pathogen immune evasion. Now, it remains to be seen if the manipulation of host cell microRNA profiles can be an opportunity for therapeutic intervention for these difficult-to-treat diseases.

## INTRODUCTION

The burden of mycobacterial infection on human health and disease simply cannot be understated, due mostly to *Mycobacterium tuberculosis*, the etiological agent of tuberculosis. Globally, it is estimated that two billion people are infected with *M. tuberculosis,* of which 10% develop active tuberculosis resulting in nearly 1.4 million deaths per year. Although there is currently a vaccine for tuberculosis, the attenuated *M. bovis* strain bacille calmette-guerin (BCG), it is considered to be largely ineffective (Orme, 2013). Another species of *Mycobacterium* that has made a significant impact on human health is *M. leprae*, the etiological agent of leprosy. Also known as Hansen’s disease, leprosy has affected human history for thousands of years both medically and sociologically due to the social stigma associated with those aﬄicted, at times resulting in forced segregation of infected individuals into isolation colonies. Fortunately, according to the World Health Organization (WHO), over 14 million leprosy patients were cured in the last 20 years due to distribution of multidrug therapy treatment. Other mycobacterial species such as *M. avium* complex also cause opportunistic infections in immunocompromised individuals, which is a serious problem for the clinical management of HIV-infected patients.

Only through intensive human effort into research and clinical care have we witnessed a decrease in the disease burden caused by pathogenic mycobacteria; however, incidence of multidrug resistant and extensively drug resistant tuberculosis (MDR-TB and XDR-TB, respectively) is on the rise, and there are even reported cases of totally drug resistant tuberculosis (TDR-TB; Velayati et al., 2009). These emerging infections dictate a need for new avenues of treatment, such as host-targeted immunotherapies, which leads us to the question: how are these disease-causing mycobacterial species, *M. tuberculosis* and *M. leprae*, able to escape our immune defenses? Here we will review the studies that have examined the function of microRNAs in mycobacterial infections and what we have learned about these diseases by going beyond profiling.

## LEARNING FROM LEPROSY

**FIGURE 1:**
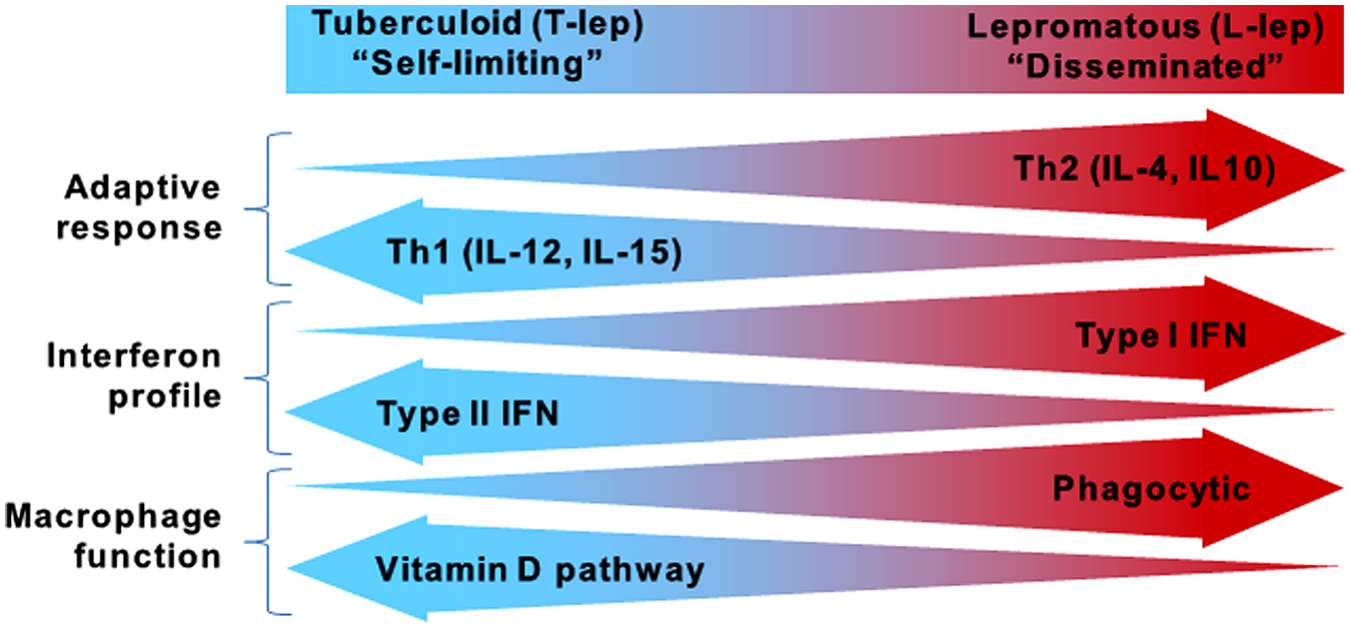
**The spectrum of leprosy.** Characteristics of the innate and adaptive immune responses in either end of the leprosy spectrum.

While the global incidence of leprosy has been dramatically reduced, research on the host defense against *M. leprae* and disease pathogenesis continues to provide insight into which immune pathways are essential for containment of mycobacterial infections. Leprosy is a powerful model to study the human immune response because it presents as a spectrum where the clinical manifestations correlate with the level of immune response to the pathogen, contributing to host defense or pathogenesis (Ridley and Jopling, 1966). At the ends of the spectrum are the tuberculoid (T-lep) and lepromatous (L-lep) forms, in which the infection is self-limited or disseminated, respectively (**Figure 1**). Unlike tuberculosis, which manifests primarily in the lungs, leprosy presents as granulomatous lesions in the skin, which can be obtained with minimal risk to the patient. Comparisons of T-lep and L-lep skin lesions have yielded important information regarding the adaptive and innate immune responses needed for protection against *M. leprae* infection. Skin lesions from T-lep patients exhibit an adaptive immune response characterized by Th1 cytokines (Salgame et al., 1991; Yamamura et al., 1991), Type II interferon (IFN) profile (Teles et al., 2013), and an innate immune response characterized by macrophages programmed to express the vitamin D-mediated antimicrobial pathway (Montoya et al., 2009). In contrast, L-lep lesions are typified by an adaptive immune response characterized by Th2 cytokines (Salgame et al., 1991; Yamamura et al., 1991), Type I IFN profile (Teles et al., 2013), and an innate immune response characterized by macrophages programmed to express enhanced phagocytic activity (Montoya et al., 2009).

## microRNAs IN LEPROSY LESIONS – STARTING WITH EXPRESSION PROFILING

As knowledge on the role of microRNAs in human conditions, such as cancer, cardiovascular disease, neurodevelopmental diseases, and autoimmune diseases continues to accumulate (Ardekani and Naeini, 2010), evidence that microRNAs may also be involved in mycobacterial infections is similarly building. Expression profiling experiments revealed 13 microRNAs were more highly expressed in L-lep vs. T-lep lesions; in contrast, only two microRNAs were more highly expressed in T-lep vs. L-lep lesions (Liu et al., 2012). mRNA targeting analysis of the microRNA species enriched in L-lep lesions demonstrated a significant preferential targeting of genes known to be important in host defense against intracellular pathogens, such as Th1-related genes (IFN-γ, IL12A, and CD40LG). In particular, hsa-mir-21, the most highly expressed microRNA in the L-lep lesions, is confirmed to target multiple genes (CYP27B1 and IL1B) in the vitamin D antimicrobial pathway, a key pathway in macrophage antimicrobial defense against mycobacterial infection (Liu et al., 2006, 2012). To understand how hsa-mir-21 can influence the outcome of mycobacterial infection, we will first discuss the role of the vitamin D pathway in macrophage antimicrobial defense.

## THE VITAMIN D ANTIMICROBIAL PATHWAY AND MYCOBACTERIAL INFECTION

Detection of *M. leprae* (Krutzik et al., 2003) and *M. tuberculosis* (Brightbill et al., 1999) by innate immune cells such as monocytes and macrophages is mediated, in part, by the pattern recognition receptor heterodimer, Toll-like receptor 2 and 1 (TLR2/1). Activation of TLR2/1 on monocytes results in the induction of the vitamin D receptor (VDR) and CYP27B1 in an IL-15-dependent manner (Liu et al., 2006; Krutzik et al., 2008). The CYP27B1 gene product (CYP27b1), a cytochrome P450 hydroxylase, is responsible for the conversion of the circulating prohormone form of vitamin D (25-hydroxyvitamin D3, 25D) into its active hormone form (1,25α-dihydroxyvitamin D, 1,25D). If the extracellular concentration of 25D is sufficient, CYP27b1 will convert 25D into 1,25D, resulting in activation of the VDR and expression of antimicrobial peptides cathelicidin (CAMP) and human beta defensin-2 (DEFB4; Liu et al., 2009).

Convergence of IL-1β signaling and vitamin D transcriptional activation is required for the TLR-induced expression of DEFB4 (Liu et al., 2009). Triggering of TLR2/1 was found to modulate IL-1β activity and increase the cell’s responsiveness to IL-1β by simultaneously (i) inducing IL-1β secretion, (ii) increasing expression of cell surface IL-1 receptor 1 (IL-1R1), and (iii) decreasing the baseline secretion of IL-1 receptor antagonist (IL-1RA; Liu et al., 2009). Loss of CAMP or DEFB4 expression, as well as blockage of the vitamin D pathway, ablated the TLR2/1-induced antimicrobial activity, implicating the vitamin D antimicrobial pathway as a critical part of the innate immune response against *M. tuberculosis* (Liu et al., 2006, 2009). Interestingly, although TLR2/1 activation of monocytes induces antimicrobial activity against *M. tuberculosis* infection (Thoma-Uszynski et al., 2001; Liu et al., 2006, 2012), it was insufficient to trigger antimicrobial activity against *M. leprae* infection (Liu et al., 2012). The differential ability of TLR2/1 activation to induce antimicrobial activity against *M. tuberculosis* or *M. leprae* negatively correlated with the induction of hsa-mir-21 during infection, where *M. leprae* induced hsa-mir-21, but *M. tuberculosis* did not (Liu et al., 2012). When considered together with the distinct microRNA expression profiles in leprosy lesions, these data are highly suggestive that infection-induced microRNA expression could regulate the macrophage innate immune response to the pathogen as a potential evasion mechanism.

## THE ROLE OF hsa-mir-21 IN ANTIMICROBIAL ACTIVITY AGAINST MYCOBACTERIA

The ability of hsa-mir-21 to alter the outcome of infection was confirmed through functional studies. Transfection of hsa-mir-21 into human monocytes results in the reduction of TLR2/1-stimulated expression of IL1B, CYP27B1, CAMP and DEFB4 and enhancement of the TLR2/1-stimulated immune-inhibitory cytokine, IL10. Conversely, knockdown of hsa-mir-21 during *M. leprae* infection of human monocytes results in enhanced expression of IL1B, CYP27B1, CAMP and DEFB4, as well as a decrease in IL10 (Liu et al., 2012). One effect of the cumulative hsa-mir-21-mediated regulation of the cellular gene expression was ultimately control of the TLR2/1 antimicrobial activity against the infection. The TLR2/1-triggered antimicrobial activity against *M. tuberculosis* was ablated by hsa-mir-21; in contrast, blocking hsa-mir-21 during *M. leprae* infection resulted in rescue of TLR2/1-induced antimicrobial activity. In addition, miR-21 inhibits IL-12p35 expression, a subunit in a major Th1 driving cytokine (Lu et al., 2009), and enhances IL-10, a major Th2 driving cytokine (Sheedy et al., 2010; Liu et al., 2012). By inhibiting the vitamin D antimicrobial pathway and skewing the Th1 vs. Th2 T-cell response in leprosy, hsa-mir-21 could be a major determinant of the clinical presentation of an infected individual.

## THE ROLE OF microRNAs DURING MYCOBACTERIAL INFECTION OF MACROPHAGES

The clinical presentation of tuberculosis, like leprosy, can be considered a spectrum with active disease and latent infection at the opposing ends of the spectrum (Wu et al., 2012b). However, comparing polar ends of the tuberculosis spectrum at the site of infection is immensely difficult due to the invasive nature of collecting lung tissue, especially from latently infected individuals. An alternative approach is to compare infection by virulent and non-virulent mycobacteria or infected and uninfected cells using *in vitro* and *in vivo* systems. Human macrophages infected with virulent *M. tuberculosis* yields high hsa-miR-125b and low hsa-miR-155 expression when compared to infection with the non-virulent *M. smegmatis* (Tili et al., 2007; Rajaram et al., 2011). This dichotomy of microRNA expression influences the induction of TNF-α, given that miR-125b binds and destabilizes TNF-α mRNA (Tili et al., 2007), whereas miR-155 enhances TNF-α production (O’Connell et al., 2009; Bala et al., 2011). Furthermore, transfection of mouse macrophages with miR-155 results in decreased *M. tuberculosis* intracellular survival (Kumar et al., 2012). It is possible that miR-155 alters antimicrobial activity through regulation of two critical processes for protective immunity: macrophage apoptosis (Ghorpade et al., 2012) and autophagy (Wang et al., 2013).

Another microRNA, miR-142-3p, is also differentially regulated by virulent and non-virulent mycobacteria. When comparing *M. tuberculosis* to *M. smegmatis* infection, miR-142-3p is up regulated rapidly (peaking at 1 h for mouse macrophages and 4 h for human macrophages; Bettencourt et al., 2013), but down regulated at a later stage of the infection (24 h post-infection in mouse macrophages; Martinez et al., 2013). Early induction of miR-142-3p interfered with phagocytosis, a required cellular process for the macrophage antimicrobial response, via targeting the mRNA of the actin binding protein N-WASP (Bettencourt et al., 2013). This disparity between regulation of miR-142-3p at early and late time points by *M. tuberculosis* may be a reflection of the pathogen’s ability to elicit microRNAs that counteract the sequential stages of the macrophage response. A separate study supports the hypothesis that time-sensitive regulation of microRNA can have a significant impact on the outcome of infection by showing that in contrast to virulent *M. tuberculosis*, infection with the non-virulent *M. bovis* BCG results in early down regulation of mmu-miR-142-3p (Xu et al., 2013). Decreased expression of mmu-miR-142-3p leads to increased expression of target gene IRAK-1, an inhibitor of TLR signaling (Ghorpade et al., 2012). Therefore, the loss of miR-142-3p would enhance recognition of the infection and induction of antimicrobial activity for *M. bovis* BCG compared to *M. tuberculosis* infection.

While control of the TLR response early during infection may impair detection thus benefiting the pathogen, multiple microRNAs targeting TLR signaling are also induced later in infection. *M. bovis* BCG infection of mice results in enhanced expression of mmu-miR-31, mmu-miR-150, and mmu-miR-146a. In contrast, the human homolog of the same microRNAs are significantly lower in the peripheral blood mononuclear cells (PBMCs) of tuberculosis patients compared to healthy controls (Ghorpade et al., 2013), suggesting a role for these microRNAs in control of chronic inflammation. Through targeting the TLR signaling cascade, miR-31, miR-150, and mir-146a (Taganov et al., 2006; Ghorpade et al., 2013) may dampen the uncontrolled inflammation that leads to tissue damage, a major cause of pulmonary morbidity and mortality in tuberculosis (Lazar-Molnar et al., 2010). Thus, the microRNA profile of the macrophage during and after infection can have a significant impact on the outcome of infection and disease pathogenesis based on the immune pathways targeted by the regulated microRNAs.

## THE ROLE OF microRNAs DURING MYCOBACTERIAL INFECTION – MORE THAN JUST MACROPHAGES

While macrophages are the target cell of mycobacterial infection, they are not the only immune cell type involved and influenced by microRNAs during infection. Critical at the interface of the innate and adaptive immune responses, the dendritic cell (DC) has the ability to activate and polarize T-cell responses, which is subject to regulation by microRNAs. Induction of mir-99b by infection of DCs with *M. tuberculosis* inhibits TNF-α production, whereas knockdown of mir-99b leads to a decrease in bacterial burden (Singh et al., 2013). Furthermore, infection of mouse DCs with *M. bovis* BCG results in higher levels of miR-146a compared to *M. tuberculosis*, thereby inhibiting the DC’s ability to differentiate Th17 T-cells, an essential component for optimal vaccine efficacy (Chatterjee et al., 2011).

T-cells are also influenced by microRNAs during infection despite not being permissive cellular hosts for mycobacteria. Infection of mice with *M. bovis* BCG results in decreased expression of mmu-miR-29 in T-cells which targets and down regulates the critical Th1 cytokine IFN gamma (IFN-γ; Ma et al., 2011). Ablation of miR-29 in mice renders them more resistant to both *M. bovis* BCG and *M. tuberculosis* infections (Ma et al., 2011), suggesting that induction of miR-29 in T-cells during infection is a facilitator of bacterial virulence. There is also evidence that hsa-miR-181a may influence T-cell responses during leprosy due to its ability to dampen T-cell activation (Li et al., 2007). For that reason, lower levels of hsa-miR-181a in T-cells derived from lepromatous vs. tuberculoid patients (Kumar et al., 2011) may explain the prevalence of T-cell hyporesponsiveness in lepromatous leprosy patients, a hallmark of disease progression (Kumar et al., 2011). Based on these studies, it is apparent that microRNA regulation during mycobacterial infection can affect multiple cell types and have wide ranging effects on the systemic immune system by modulating both the innate and adaptive immune responses to the pathogen.

## REGULATION OF GENE EXPRESSION PROFILES IN *M. avium* INFECTION BY microRNAs

While the studies above unequivocally support the idea that a single microRNA regulated during mycobacterial infection can influence the resulting immune response by targeting key genes, it was unclear whether the overall microRNA profile and its cumulative targets could alter the gene expression profile of the infected cell in a concerted manner. An integrated microRNA-mRNA analysis of infected human macrophages helped provide insight into the cumulative impact of microRNA regulation during infection. MicroRNA and mRNA expression profiles of macrophages infected with *M. avium* subsp. *hominissuis* were obtained, and microRNAs and mRNAs with negatively correlated expressions were mapped onto a gene network and overlapped with target predictions (Sharbati et al., 2011). The analysis revealed multiple immune pathways potentially targeted by the conglomeration of multiple infection-induced microRNAs, including: apoptosis, cytokine and inflammatory response, and NF-κB activation (Sharbati et al., 2011).

There appears to be a concerted regulation of the apoptosis pathway during *M. avium* subsp. *hominissuis* infection. Two microRNAs induced during infection, hsa-miR-29a and let-7e, target and inhibit the pro-apoptotic caspase 3 and caspase 7 networks (Sharbati et al., 2011). Down regulation of these caspase pathways promotes an anti-apoptotic macrophage state, which is beneficial for bacterial survival. In addition, the pro-apoptotic tumor suppressor protein p53 (TP53) is potentially targeted by let-7e and miR-886-5p, microRNAs also up regulated during *M. avium* subsp. *hominissuis* infection (Sharbati et al., 2011). Whether regulation of TP53 expression through microRNA expression during infection can impact apoptosis remains to be seen. Taken together the data from this study indicates that the global microRNA profile induced during infection could dictate the gene expression profile of the host cell. Hence, it would be of benefit for pathogens to induce microRNAs that target immune pathways and down regulate microRNAs that perpetuate the immune response to facilitate immune evasion during infection.

## microRNA SIGNATURES IN TUBERCULOSIS

There has been significant interest in identifying a microRNA signature of tuberculosis to provide diagnostic biomarkers and new avenues for studying the immune response. Multiple microarrays have been performed on samples derived from tuberculosis patients, ranging from PBMCs, pleural fluid mononuclear cells (PFMCs), pooled serum, and even PBMCs stimulated with mycobacteria or mycobacterial ligands *ex vivo*. While a definitive microRNA signature has yet to be determined, three microRNAs with higher expression in active tuberculosis patients compared to healthy controls have been positively evaluated for use as a biomarker: miR-155 and miR-155* in stimulated PBMCs (Wu et al., 2012a) and miR-29a in pooled serum (Fu et al., 2011). In addition to expression analysis, microRNA single nucleotide polymorphism (SNP) analysis has revealed a correlation between SNPs in miR-146a (rs2910164) and miR-499 (rs3746444) and increased pulmonary tuberculosis susceptibility in certain populations (Li et al., 2011). More work is required before microRNAs can be integrated into clinical diagnostics and care for tuberculosis patients.

## CONCLUSION

The studies presented here support a role for regulation of microRNA in mycobacterial infection and disease. Differential expression of microRNAs in self-limiting vs. progressive infections or non-virulent vs. virulent infections illustrates the ability of specific microRNAs present during infection to influence the outcome of infection (Summarized in **Table 1**). Factors that determine whether the microRNA expression profile during infection favors or targets the immune response when the host cell and *Mycobacterium* are engaged could be a key point in determining the outcome of the infection. As more insight is gathered on (i) the functional consequences of microRNAs regulation during mycobacterial infections, (ii) how the genes and pathways they target facilitate the immune response and iii) how they are regulated by the pathogens, an opportunity for host microRNA-directed therapeutic intervention may present itself.

**Table 1:** Summary of microRNAs and their impact during infection.

microRNA	Cell type	Expression level	Target(s)	Function
hsa-mir-21 (human)	Skin lesions, macrophages, monocytes	↑ L-lep vs. T-lep lesion ↑*M. leprae* infection	CYP27B1, IL1B, IL-12p35, PDCD4	Inhibition of antimicrobial activity
hsa-miR-125b (human)	Macrophages	↑ *M. tuberculosis* vs. *M. smegmatis* infection	TNF-α	Possible subversion of host immunity
miR-155 (human and mouse)	Macrophages	↑ *M. smegmatis* vs. *M. tuberculosis* infection	SHIP1	Enhancement of TNF-α, and decreased *M. tb* intracellular survival
miR-142-3p (human and mouse)	Macrophages	↑ *M. tuberculosis* vs. *M. smegmatis* infection (early) ↓ BCG vs. *M. tuberculosis* infection (late)	N-WASP, IRAK-1	Disruption of phagocytosis, altered TLR signaling
miR-99b (human and mouse)	Dendritic cells	↑*M. tuberculosis* infection	TNF-α	Increased bacterial burden
miR-146a (mouse)	Dendritic cells	↑ BCG vs. *M. tuberculosis* infection	IRAK-1, TRAF6	Suppression of IL-6, IL-1B, and TNF-α expression and inability to promote Th17 cells
miR-29 (mouse)	T cells	↓ BCG-infected mice	IFN-γ	Regulation of Th1 responses
hsa-miR-181a (human)	T cells	↓ T-lep vs. L-lep patients	SHP2	Dampens T cell activation
hsa-miR-29a (human)	Macrophages	↑*M. avium* infection	Caspase 3	Promotes an anti-apoptotic state
hsa-let-7e (human)	Macrophages	↑*M. avium* infection	Caspase 7	Promotes an anti-apoptotic state

## Conflict of Interest Statement

The authors declare that the research was conducted in the absence of any commercial or financial relationships that could be construed as a potential conflict of interest.
